# Setting Up a Just and Fair ICU Triage Process during a Pandemic: A Systematic Review

**DOI:** 10.3390/healthcare12020146

**Published:** 2024-01-08

**Authors:** Rhyddhi Chakraborty, Nebil Achour

**Affiliations:** School of Allied Health and Social Care, Anglia Ruskin University, Cambridge CB1 1PT, UK; nebil.achour@aru.ac.uk

**Keywords:** COVID-19, ethics, ICU, pandemic, prioritisation, South Asia

## Abstract

Introduction: Triage is a dynamic and complex decision-making process to determine fair access to medical care in mass casualty situations. Triage takes place through healthcare settings including Intensive Care Units (ICUs). Triage governing principles have been subject to ethical debates for a long time specifically with the recent global pandemic of COVID-19. This study aims to revisit the ethical principles guiding patient prioritisation during recent COVID-19 disaster triage in the Indian subcontinent and attempts to look for principles with consideration of social justice. Methods: Key electronic databases such as WHO, EMBASE, and DOAJ were used to access published literature relating to ICU triage in the Indian subcontinent. Literature on and from 2015–2022 were included in this study. The SPICE framework was used to identify the literature. The Inclusion criteria were as follows: Literature with ethical connotations focusing on India and neighbouring countries, and in an ICU setting during pandemics. The Exclusion criteria were as follows: Literature focusing on other countries, without ethical foundations, hospital admissions, and non-COVID-19 ICU admissions. The PRISMA standard was applied to screen the appropriate literature. The BOOLEAN operator “OR” was used to enhance the literature search. Finally, six papers were found suitable for this study and thus were included in the literature review. Additionally, for the second time, the frequency of certain ethical phrases was reassessed in the plans and guidelines to check the changed awareness of ethical pandemic planning, if any. A thematic analysis was applied to analyse the data and generate findings and new knowledge. Results: The findings highlight gaps in knowledge around ICU triaging in the region which indicates the scope of better ethical pandemic preparation at the regional level. The findings show that there is a debate between researchers on prioritisation from available resources and ethical perspectives and principles associated with fair access to healthcare even during pandemic times. The literature also highlights enhancing the regional capacity and building equitable approaches to reduce existing health inequities and the need of the social justice framework for ICU triaging during a pandemic. Conclusions: ICU triaging in five South Asian neighbour nations was studied for the presence of a guided ethical framework. Additionally, for the second time, certain ethical phrases were reassessed in the plans and guidelines; however, usage of those terms was found to be significantly low. The discussion shows that the plans and guidelines have the scope to improve ethical ICU triaging in these countries and in the specific region. After analysing different ethical guidelines, this study emphasises that there is a need for a just and fair framework, specifically a social justice framework in ICU triage in the subcontinent to address the underlying health inequities.

## 1. Introduction

The literature on pandemic planning and responses suggests that response to public health emergencies should be guided by specific ethical norms and considerations, in addition to medical and military logistical skills [1,2]. Lessons learned from past pandemics and epidemics such as SARS (2003) have demonstrated the reasons for and ways in which incorporating ethical concerns into pandemic plans and policies may have made and can still make a substantial impact in resolving many ethical issues that a pandemic crisis may present. Researchers have argued that there are ethical concerns identified in almost every aspect of pandemic planning, including allocating healthcare resources, prioritising the distribution of services, resolving conflicts between patient and community rights, and balancing healthcare workers’ professional and personal obligations. The World Health Organisation’s [3] guidance document “Ethical considerations in developing a public health response to pandemic influenza” was created to help member states include ethical values and considerations in pandemic planning. There were several alleged advantages of incorporating moral principles into pandemic response plans, from lowering death rates to increasing public collaboration [1,4]. Nonetheless, a study demonstrated that differential pandemic planning existed, and ethical value inclusion also varied between countries; some made notable efforts to include ethical principles in their pandemic plans and policies, while others lagged behind [5].

The study conducted from 2009 to 2011 [1] in the context of South Asian nations, specifically India, Nepal, Bangladesh, and Sri Lanka, revealed that while many nations had actually been able to put together a national pandemic plan or draft during the last pandemic, the use of ethical language and the process of including ethical values or considerations in their pandemic plans and policies were at an early stage. India, for instance, experienced A H5N1 (Avian Influenza, 2006–2006) and A H1N1 (Human Influenza, 2009) and created two plans: the *Influenza Pandemic Preparedness and Response* Plan to mitigate avian influenza in 2005 and the *Action Plan, Pandemic Preparedness and Response for Managing Novel Influenza (AH1N1) (earlier called Swine flu) (or that arising from any other novel strain of Influenza*) in 2009. However, a study revealed that India used shockingly little ethical language, far less than some of its much smaller bordering countries. Additionally, both the Indian pandemic plans [2005, 2009] acknowledged the livelihood risks and the vulnerability of the immune-compromised people, but had the oversight of the Social Determinants of Health (SDH), and the heightened risk of excess morbidity and mortality due to the SDH among certain social and economic strata of the society during the pandemics [1]. The 2009–2011 study maintained that within these lacunae lay the scope of health inequities, which needed to be addressed urgently. It argued that in order to address these lacunae, and particularly to address the need for extending equitable protection to those who may need it most, pandemic planning in India should take guidance from the ethical precepts of social justice theories. With this backdrop, the present study revisits the ethical principles guiding patient prioritisation during disaster triage in the Indian subcontinent and attempts to look for triaging principles with consideration of social justice in the Indian subcontinent.

Triage is a dynamic and complex decision-making process to prioritise access to medical care, both healthcare treatments and hospital care. It involves an ethical element to secure fair access to available healthcare facilities and resources and has been the subject of many debates [6]. Intensive Care Units (ICUs) might be considered as free from triage due to the sensitivity and criticality of patient conditions; however, triage was actually conducted in ICUs during COVID-19 pandemic where these units were overwhelmed with critical cases and resources were limited despite the pandemic preparedness of countries [7]. A deeper look into the ICU triaging principles revealed that countries developed ICU guidelines to deal with mass casualties and with a great variance in principles: saving the maximum number of lives through medical prognosis, adopting age, life expectancy, quality of life, etc. [7]. This variance had been a concern for some researchers as the adopted triage process had overlooked the existing health inequities and contributed to widening the health gap in societies [8].

With the backdrop of COVID-19 in the Indian subcontinent, this study revisits the ethical pandemic planning in the subcontinent with specific focus on the guidelines and guiding ethical principles for ICU triaging.

## 2. Methodology and Protocol

This study is founded on qualitative research based on secondary data. The literature was searched for using the following: (a) electronic databases (see Table 1); (b) reference lists of included articles; (c) national and regional pandemic guidelines and policies; (d) ministry/department of health websites of India, Nepal, Bangladesh, Sri Lanka, Pakistan, and Bhutan (the Bhutan plan was finally excluded for non-availability on the website) for pandemic preparedness documents; and (e) grey literature [9,10,11,12,13,14].

Search keywords included but were not limited to “COVID 19”; “Critical Care”; “Ethics”; “ICU”; “Pandemic”; “Prioritisation”; “Rationing”; “Social Justice”; “Sub-continent”; and “Triage” and included Boolean operators “AND” and “OR”.

**Figure 1:**
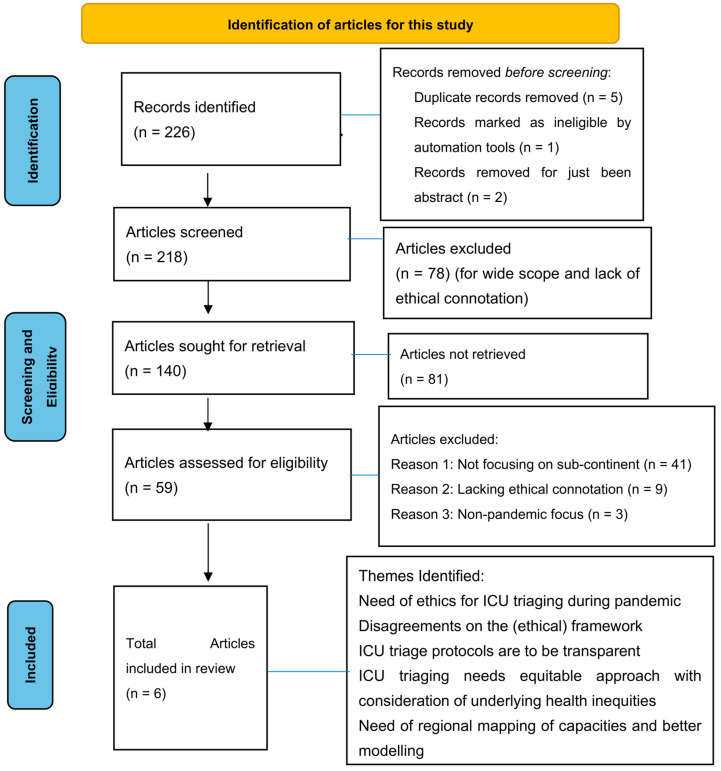
PRISMA flow diagramme.

Setting (S), Population (Perspective), Intervention (I), Comparator (C), Evaluation (E) or the SPICE framework was used to identify the literature. Preferred Studying Items for Systematic Reviews [15,16] and Meta Analysis (PRISMA) (Figure 1) standard was applied to screen the appropriate literature. The relevant literature search has been limited to only English language articles published between 2015–2022; literature with explicit mention of ethical guidelines used for pandemic triage was included. The initial search identified 226 articles and after initial screening, 140 articles were included in this study. The application of inclusion and exclusion criteria led to 59 articles which were then included for eligibility assessment. With the removal of duplicates, 40 articles were included after the initial screening (Table 1).

The final assessment, after screening and eligibility check, resulted in *n* = 6 with *n* = 53 being excluded for not having a focus on Indian subcontinent and/or not referring to ethical ICU triage as shown in Figure 1.

## 3. Data Abstraction and Synthesis

Data were abstracted, drafted, and refined. Two kinds of methods were used for data abstraction: (a) identifying the literature prioritising the ICU triage process in the South Asian context and conducting a critical appraisal, and (b) reviewing the ethical contents in the country-specific documents as sourced from the governmental websites. The tables were cross checked twice to achieve consistency and to add missing information (e.g., Reference Location; Need of ethics for ICU triage; Ethical Framework; and Open and Transparent Protocol for ICU). Table 2 summarises this abstraction and Table 3 summarises the general critical appraisal. Since the methodology also involved the exclusion criteria, Table A1 summarises the articles with the justified usage of the exclusion criteria.

Due to the heterogeneity of data resulting from the scoping searches, a narrative (thematic) synthesis and qualitative appraisal was undertaken. Table 2 presents the summary of these.

Table 2 summarises to what extent the referred guides agree and disagree on the broad themes about ICU triage and admissions. This abstraction and broad thematic analysis were needed to proceed further with the critical appraisal and in-depth thematic analysis.

## 4. Critical Appraisal

Collected data have been appraised for methodological and quality assessment according to Appraisal of Guidelines for Research and Evaluation Instrument (AGREE II) [17]. AGREE II was adopted for critical quality appraisal of documents for (a) it is designed to assess guidelines developed by local, regional, national, or international groups or affiliated governmental organisations; (b) it is generic and can be applied to guidelines in any health or disease area targeting any step in the healthcare continuum, including those for health promotion, public health, screening, diagnosis, treatment, or intervention [18]. Scores were used on the main guidelines, policies, and plans to calculate domain scores for AGREE II. A score of 1 was used to indicate the presence of information in the guidelines (see Table 3).

Table 3 summarises the critical appraisal with appraiser score, and it highlights that Nepal and Pakistan guidelines score better than the other neighbouring countries.

The second approach was to review the ethical contents in the country-specific documents as sourced from official websites. A previous study [5] identified the existence of few ethical terms in the South Asian influenza pandemic plans. This study further investigates if those ethical terms/expressions have been used to deal with the ICU triage process during the COVID-19 pandemic and if there is any enhanced awareness and consideration of the social justice framework. Table A5 summarises the findings from this study, whereas previous findings have been reported in Table A2, Table A3 and Table A4. Sentences containing the ethical phrases of concern were extracted first. They were further investigated to see if the terms were employed with an ethical meaning in their respective contexts. That is, in addition to a mechanical key term search, the usage of the terms was subjected to a semantic assessment to determine whether or not it has ethical value in its particular context. When no such significance was discovered, this was also reported in this study. The analysis also omitted partial occurrences of terms in separate words. The terms “moral” in the “femoral”, “fair” in the “affair”, “ICU” in “difficulties”, and “open” in “propensity”, for example, were eliminated from this study, in addition to phrases with primarily medical/clinical implications. For instance, “exhaled air spreads through…” was omitted. Extracted phrases were appraised for their ethical meanings for further analysis. As mentioned above, Table A5 shows this study’s findings as well as the frequency with which the term was employed.

## 5. Findings

The PRISMA flow diagramme (Figure 1) illustrates the process of selection and identification of articles. Of the articles, one concerned ethical decision making for ICU triage, one is concerned with lessons learned from COVID-19, and one is concerned with the containment plan. At least eight of these were guidelines at the national level and were produced by government bodies; one was produced by the Bioethics Commission. Narrative synthesis identified seven broad themes across these articles and guidelines, which are summarised in Table 2. As Table 2 summarises, while country documents mentioned the need for open and transparent ICU protocols, no guideline demonstrated complete coverage of all themes. However, the Nepal guidelines are relatively better at matching the themes than the others. Results specific to each theme are described below.

No guidelines and/or articles were excluded for their quality. However, from the quality assessment perspective, the Nepal and Pakistan guidelines scored better than the others for significantly specifying different stakeholders to be involved in decision making and the implementation stage. While documents were prepared by the governments, only Pakistan’s document was published by the National Bioethics Committee and the Sri Lankan document was prepared in collaboration with the physicians as shown in the documents.

### 5.1. Theme 1: Need of Ethics for ICU Triaging during Pandemics

In the context of the need of ethics for ICU triaging, there are areas of agreement in the literature that indicate that scarce medical resources of the ICU such as beds, ventilators, etc. are to be distributed fairly and reasonably during a pandemic [19,20,21]. Literature also specifies that resources are to be distributed fairly and reasonably to make them accessible to all. However, there is limited understanding of standard ethical principles in ICU triage decision making, especially in South Asian countries [20]. Out of the five countries, only the Pakistan guidelines explicitly mention the need of ethical principles for ICU triaging during pandemics like COVID-19 ([22], p. 4). Nepal’s plan, on the other hand, implicitly indicates how differential groups such as migrants, people with co-morbidities, etc. are not to be overlooked for disease screening, but these undermined groups have been mentioned only for the public health screening and not for the ICU admission protocol ([23], p. 46). Hence, no consistent awareness or concern of the need of ethics for ICU triaging has been noticed in the South Asian guidelines.

### 5.2. Theme 2: Disagreements on the (Ethical) Framework

There is disagreement on the rationale principle behind the just allocation during the crisis [24,25,26,27,28]. A requirement of strengthened national guidance for the protocol and policy for ICU triaging [29] was highlighted. To conduct a deeper assessment, ICU triage frameworks for the South Asian countries were revisited.

In general, a variety of criteria were given for ICU admissions to inform decisions on who should be admitted to the ICU based on the parameters of respiratory rate, SPO2 level, probability of survival, co-morbidities, and age. All of the guidelines agreed that components of these characteristics should be utilised in combination in ICU admission decision making, and the onus remains on the clinical teams led by physicians to assess the final admission criteria for individual patients. However, when it comes to an ethical ICU triage process, it is only Pakistan’s guidelines which mention using a compassionate, respectful, and empathetic approach ([22], p. 5). The Nepal guidelines specify different socioeconomically vulnerable groups, but these groups have been undermined in the context of ICU admission and treatment. Hence, no consistent guideline on ethical triaging or need of ethical framework for ICU triaging has been found in the relevant South Asian documents.

### 5.3. Theme 3: ICU Triage Protocols Are to Be Transparent

ICU triage protocols are expected to be transparent, built upon trust, to be inclusive, and to include public health values [24]. Multiple ethical values need to be balanced for various interventions and circumstances to develop prioritisation guidelines and standard operating protocols [30]. There are limited data which support advanced ethical consultation and reflection to make the process more inclusive and value-based in the South Asian guidelines and plans. The importance of open and transparent information sharing had been hinted at by the Nepal and Pakistan guidelines (Table A5). The Pakistan guideline, specifically, highlights that Standard Operating Procedures (SOPs) need to be more coherent ([22], p. 4). On the other hand, the Bangladesh plan used the term “open” but with different connotations, such as “Do not go near any open flames when using oxygen…” ([31], p. 33). Hence, no indication of clear, transparent, and standardised ethical ICU protocol could be identified in the plans and guidelines.

### 5.4. Theme 4: ICU Triaging Needs Equitable Approach with Consideration of Underlying Health Inequities

ICU triaging needs an equitable consideration safeguarding the right to health of all [32]. However, the main and generic focus of equity has been utilitarian– saving maximum lives; lottery-based rationing thereby overlooks the underlying health inequities. Hence, a just and fair approach with consideration of an equitable framework is desirable for the ICU triage decision-making during pandemics. In this context, only Nepal’s guidelines have mentioned equity (Table A5), but this has not been extended to set the criteria for ICU admissions and treatment.

Four fundamental ethical values, obtained from previous pandemic models, are usually popular in ICU triaging: Maximising the benefits produced by scarce resources; Treating people equally; Promoting and rewarding instrumental value; Giving priority to the worst off [30]. In some plans and guidelines of South Asian countries, although these concepts have been used, they have been used for different contexts, such as for general health care and public health concerns, but not specifically for ICU triaging. The Pakistan plan, though, encourages not overlooking other patients for ICU admissions; in all countries, the ICU admission criteria are founded on the basis of medical conditions, overlooking socioeconomic and health inequities. Hence, more research is needed to incorporate the perspective of existing health inequities in just and fair ICU rationing during pandemics.

### 5.5. Theme 5: Need of Regional Mapping of Capacities and Better Modelling

With lessons learnt from past pandemics, the literature recommends maintaining a central database of ICU resources in order to evaluate health system performance, both within and between countries, which may help to develop related health policy [33]. Regional modelling is needed to cope with the pandemic pressure for the ICU [24,34]. As a pandemic respects no borders, ICUs of the region can also be overwhelmed at the same time with no capacity to transfer patients and COVID-19 has shown evidence of this. While Nepal mentions inter-country and regional collaboration (Table A5), nothing has been remarkably highlighted for mapping regional capacities in any other plans. Hence, there is a need to have a better insight into the regional ICU triaging process.

Recently, COVID-19 has highlighted that pandemic preparedness, including ICU preparation in a South Asian context, needs to be founded on a better framework [24,34]. However, there is an oversight of the framework recommendation with insights of underlying health inequities. Therefore, there is a need for research to revisit the framework recommendation, which is founded on equitable rationalisation.

In addition to these thematic analyses, inclusion of ethical terms/expressions were searched for in the South Asian guideline and plans for the second time. In general, the usage of ethical language in the guidelines and plans, once again, is said to be low. None of the plans, except Pakistan’s, has a separate section on ethical considerations; whatever ethical terms have been found are used as part of the content of the plan in general.

Out of the 18 terms searched for, the common terms used for COVID-19 are *communication*, *protection,* and *responsibility*. The terms which have not been found in any COVID-19 plans’ guidance are *Accountability*, *Fair*/*Fairness*, and *Responsiveness*.

*Collaboration* signifies working together. However, it is only the Nepal and Pakistan guidelines which mention intersectoral collaboration and collaborative decisions.

*Ethics*, the other term, appears again in Nepal’s and Pakistan’s guidelines which use the term to promote better public health interventions and the rational allocation of healthcare resources.

The Nepal plan has shown concern for equity but for public health measures, not particularly for ICU triage. It has also used strategies to boost the *morale* of the healthcare workers. It is the only plan to use the context of *rights* (human rights).

*Minimising risk of transmission* was the most used expression in the Sri Lanka plan and has been used to designate areas to prevent a COVID-19 spread.

The expression *Reasonableness* was used in the Bangladesh plan and has been used in the context of rationale imagination but not in relation to reasonable ICU allocation.

*Representation/represent* was used in the Nepal, Sri Lanka, and India plans to indicate intersectoral representations in the COVID-19 prevention team. However, no representations of the vulnerable groups have been mentioned in any documents.

Finally, the context of *Transparency* and *Trust* were mentioned in the Nepal and Pakistan guidelines to indicate open sharing of information and building rapport among the teams and community. Importantly, although the COVID-19 pandemic led to many issues and challenges in relation to the ICU, with the exception of the Pakistan guidelines, no other plans use the term ICU *prioritisation*, though they discuss vaccination strategies.

## 6. Discussion

This study reveals several traits which may contribute to regional pandemic planning and ethical ICU triage in a South Asian context. From the synthesis and analysis of this study, it can be said that the guidelines and plans prepared by the five South Asian countries have been rapid with less or no consideration given to the existing socioeconomic determinants, health inequities, and social justice. Neither has this been observed in the deciding factors of ICU triage in the individual countries.

The similarity between the countries is mainly shown by setting up the medical assessments for ICU admissions. Regarding ethics, once again, the usage of expressions is low. While this portrays the individual and collective effort to be ethically concerned and to be aware of pandemic situations, it also implies that these countries do not appear to be adequately and ethically prepared to address pandemic-like disasters. Relatively, Nepal and Pakistan used more ethical terms than others. However, for regional pandemic preparedness, individual countries should have been better ethically prepared for COVID-19. The plans have also not explicitly mentioned, or implicitly suggested, the need for any ethical framework, or values, as guiding principles for ethical ICU decision making as part of pandemic preparedness, especially, involving socioeconomically vulnerable communities in these countries. There is also no overt attempt to identify probable major ethical issues, such as how to ensure ‘fair’ access to a limited number of ICUs. There is also no special effort visible in any of the plans to indicate procedural guidelines about how to ensure that ICU decision making could be ethically sound involving more stakeholders.

In certain circumstances, an ethical word is used, but the usage does not appear to be properly thought through. For example, the word “protection” appears quite frequently. However, none of the strategies emphasise the significance of protecting socioeconomically vulnerable individuals. It is also worth noting that none of the plans mention the ethical word “fairness”, despite the region’s great diversity in religious, cultural, and political beliefs, as well as noticeable socioeconomic inequities. Apart from a huge population living below the poverty line, the majority of the countries in question also have a sizable marginal population, which includes minorities, tribes, and nomads. There should have been better efforts to indicate how the vulnerability of the marginal people will be protected during a pandemic, especially in the context of ICU triage by incorporating the social justice framework in pandemic guidelines and plans of the subcontinent.

## 7. Conclusions and Recommendations

ICU triaging in five South Asian neighbouring nations was studied for the presence of a guided ethical framework. Additionally, for the second time, certain ethical phrases were reassessed in the plans and guidelines, and usage of those terms was found to be significantly low. A discussion shows that the plans and guidelines have the scope to improve the ethical ICU triaging in the countries and in the region. After analysing different ethical guidelines, this study emphasises that there is a need for a just and fair framework, mainly a social justice-based framework, in ICU triage in the subcontinent to address the existing health inequities.

In the crisis hours of a pandemic, an open and transparent ethical ICU triage can help avert many irrational strategies impacting the service delivery and can also save many unwanted and premature deaths. Inequalities in society are unavoidable. However, if they are thoughtfully incorporated in the pandemic planning, many real-time disasters can be averted. And for this, more research, with a vision of social justice, is needed in this domain.

### Limitations

This study has the limitation to conduct the systematic review on the secondary literature and grey literature. Additionally, the present work is disclosure-based and thus is limited to only what is disclosed on the website of the Ministry of Health or on the official websites. No direct, extensive primary data collection has been undertaken. Moreover, this study uses a mixture of guidelines, articles, and plans to conduct the synthesis and analysis.

## Figures and Tables

**Table 1:** List of databases searched.

Database	Initial Hits	Results after Initial Screen	Key Words Used to Search Relevant Literature
PubMed/MEDLINE	10	7	“COVID 19”; OR “Critical Care”; OR “Ethics”; OR “ICU”; OR “Pandemic”; OR “Prioritisation”; OR “Rationing”; OR “Social Justice”; OR “Sub-Continent”; OR “Triage” AND year_ cluster: (“2021” OR “2020” OR “2022” OR “2019”) AND (year_ cluster: (2015 TO 2022)
COCHRANE	9	6	
WHO	5	2	
King’s Fund	6	0	
NHS	6	1	
DOH	3	2	
Nuffield Council on Bioethics	3	3	
EPPI-Centre	2	1	
Centre for Reviews and Dissemination	1	0	
BMA	10	3	
NICE	2	2	
Intensive Care Society	16	13	
ProQuest	15	15	
SCOPUS	6	6	
EUBIOS Ethics Institute	2	2	
Ministry of Health and Family Welfare, India	73	48	
Ministry of Health and Population, Nepal	6	3	
Ministry of Health, Bhutan	N/A	N/A	
Ministry of Health and Family Welfare, Bangladesh	14	3	
Ministry of Health, Sri Lanka	14	12	
Ministry of National Health Services Regulations and Coordination, Government of Pakistan	17	14	
Total	226	140	
Total following removal of duplicates and application of inclusion/exclusion criteria	59	40	

**Table 2:** Summary of references for the COVID-19 ICU admissions and allocation.

Reference Location	Need of Ethics	Ethical Framework (Explicit/Implicit)	Open and Transparent Protocol for ICU	ICU Triaging Needs Equitable Approach	Suitability of Existing Rationing Strategy for Pandemic ICU Triage	Need of Regional Mapping of Capacities	Need of Better Modelling
India	No	No	Yes	No	No	No	No
Nepal	Yes	Yes/Implicit	Yes	Yes	No	Yes	No
Pakistan	Yes	Yes	Yes	No	No	No	No
Bangladesh	No	No	Yes	No	No	No	No
Sri Lanka	No	No	Yes	No	No	No	No

**Table 3:** Summary of the quality appraisal of included articles.

References	Scope and Purpose Stated for ICU	Stakeholder Involvement Stated	Rigour of Development	Clarity of Presentation	Applicability	Editorial Independence	Appraiser Score
India	Yes	Not explicitly	Containment Plan by Govt.	Yes	Yes	Yes	5
Nepal	Yes	Yes	Lessons Learned by Govt.	Yes	Yes	Yes	6
Pakistan	Yes	Yes	National Bioethics Commission	Yes	Yes	Yes	6
Bangladesh	Yes	Not explicitly	Guideline by Govt.	Yes	Yes	Yes	5
Sri Lanka	Yes	Not explicitly	Guideline by Govt.	Yes	Yes	Yes	5

## Data Availability

The secondary data as collected for this systematic review has been presented in the appendices and has been listed in the reference list.

## Appendix A

**Table A1:** Summary of excluded research.

Author(s)	Title	Reference	Reason for Exclusion	Additional Notes
Ballantyne et al., 2020 [35]	Revisiting the equity debate in COVID-19: ICU is no panacea	No specific	Generic article	Did not focus on South Asia
Tyrrell et al., 2020 [17]	Managing intensive care admissions when there are not enough beds during the COVID-19 pandemic: a systematic review	One South Asian country and others	Only South Asian country focused on is Sri Lanka	No comparison with other South Asian countries provided
White and Lo, 2021 [8]	Mitigating Inequities and Saving Lives with ICU Triage during the COVID-19 Pandemic	No specific	Generic article	Did not focus on South Asia
Rasita et.al., 2021 [36]	Ethics of ICU triage during COVID-19	No specific	Generic article	Did not focus on South Asia

## Appendix B

**Table A2:** Ethical values and expressions in salient literature (2003–2011) (Source: Adapted from [5]).

Ethical Value Term and Expression
Accountability/Accountable
Autonomy
Collaboration
Communication
Competence

Confidentiality
Consent
Disparity
Diversity
Duty
Ethic/s

(a) Equality, (b) Egalitarian
(a) Equity, (b) Equitable
Fair/Fairness
Freedom
Justice (a) Global; (b) Social
Human Rights
Inclusiveness/Inclusive
Justice
Liberty
Minimizing harm
Moral
Neighbourliness
Non-discriminatory/ Non discrimination
Obligation
Open/Openness
Participation
Privacy
Proportional/ Proportionality
Protection
Reasonableness/Reasonable
Reciprocity
Representation
Respect
Responsibility/ Responsible
Responsive/ Responsiveness
Right/Rights

Solidarity
Stewardship
Transparency/ Transparent
Trust
Unity
Utilitarian
(a) Utility, (b) Efficiency

## Appendix C

**Table A3:** Frequency of ethical terms in Indian Influenza Pandemic Plans (Source: Adapted from [5]).

Ethical Terms/Expressions	Total No. of Mentions in Plan (DGHSa 2005)	Total No. of Mentions in Plan (DGHS 2009)	Samples of Supporting Quotations in Plan (DGHSa 2005)	Samples of Supporting Quotations in Plan (DGHS 2009)
Collaboration	4	1	“Ensure rapid virological characterization in collaboration with WHO/lead international agencies.” (pp. 18, 22, 26) “Collaborate with international agencies to determine pathogenicity to humans.” (p. 22)	“The material prepared by MOHFW in collaboration with WHO and UNICEF would be translated into vernacular languages and given to the State Governments.” (p. 26)
Communication • Risk • Outbreak/evolution of pandemic • About vaccine • About the nature of restrictions	8	26	Some selected examples: “Establish effective communication with community, health care providers and the media.” (p. 6) “Establish an effective channel of communication with key response stake holders in government, non-Govt. Public and Media.” (p. 11) “Communication–To achieve public acceptance of the event.” (p. 33)	Some selected examples: “Risk Communication would be the most important non-pharmaceutical intervention.” (p. 12) Risk Communication “Public would be made aware of the need to self-quarantine through well managed risk communication strategy using print and visual media.” (p. 23) “…the objective of the communication would be to create wide scale public awareness and sensitize communities to appropriate behaviours before pandemic.” (p. 26) “…interpersonal communication training module and aids on pandemic influenza will be developed for all grass root health workers with partners/UNICEF/WHO” (p. 27)
Minimizing harm/risk/negative impact/social disruption/economic impact	2	2	“To minimize the risk of human infection from contact with infected animals.” (p. 14) “To minimize the risk of human infection from contact with infected animals.” (p. 15)	“All technical procedures should be performed in a way that minimizes the formation of aerosols and droplets.” (p. 110) “Early implementation of infection control precautions to minimize nosocomial/household spread of disease.” (p. 118)
Representation	1	-	“Include new institutions in the network to have representation of all zones.” (p. 9)	
Trust	-	1	-	“This will help in building public trust…” (p. 27)

## Appendix D

**Table A4:** Example of usage of ethical terms in South Asian Influenza pandemic plans (Source: Adapted from [5]).

Sl. No.	Ethical Term/Expression	Nepal Plan (August 2007–November 2010)	Bangladesh Plan (2006–2008)	Bangladesh Plan (2009–2011)	Sri Lanka Plan (2005)	Sri Lanka Plan (2006)
1	Accountability	1 “…ensuring a two-way flow of information and accountability.” (p. 46)	-	-	-	
2.	Collaboration	3 Some selected examples The DLSO will in collaboration with the District Local Development Office, train in one day workshops. (p. 14) “…with collaboration from regional, district and local level sub-committees.” (p. 53)	18 One selected example “So multi-sectoral collaboration and coordination are of paramount importance…” (p. 24)	8 One selected example “Ensure essential services; and to strengthen bilateral, regional and international collaboration.” (p. 1)	14 One selected example “…all organisations including the government, private sector and community require close collaboration and synergy.” (p. 7)	3 One selected example “…in collaboration with the Estate Infrastructure and Livestock Department.” (p. 1)
3	Communication	127 Some selected examples “…improving the capacity for risk communication.” (p. 47) The Plan proposes a national communication strategy… (p. 47) The failure of a communication response in Nepal during a pandemic could result in major panic (p. 48)	39 One selected example “To establish and ensure an integrated communication strategy responsive to public concerns.” (p. 28)	72 Some selected examples “Official communication during outbreak, response and control activities” (p. 3) “Scientific communication among scientists and officials through training, workshop and meeting” (p. 3)	26 Some selected examples “…risk communication are critical steps of preparedness.” (p. 3) “Establish communication networking among all stakeholders.” (p. 36)	1 “Risk Communication– Communication Strategic Plan was developed by the UNICEF in collaboration with the Epidemiology Unit, Health Education Bureau and other stakeholders” (p. 3)
4	Ethic/s	1 “…abide by the national and international accepted ethical standards.” (p. 33)	-	-	-	-
5.	Equity	1 “…they are within an equity and human rights perspective…” (p. 33)	-	-	-	-
6	Fair/Fairness	2 “If the backyard poultry farmers are paid a fair compensation to cover the value of the birds destroyed,” (p. 17) “…Rs.100 per bird and for all birds culled from his flock, is a fair rate of compensation.” (p. 17)	-	-	-	-
7	Human Rights	1 “…they are within an equity and human rights perspective…” (p. 33)	-	-	-	-
8.	Minimizing harm/risk/negative impact/social disruption/economic impact	4 “…minimize public fear and facilitate public protection…” (p. 16) “The plan is developed to minimize the risks…” (p. 48) “…minimize the social disruption…” (p. 52)	7 Some selected examples “…to minimize the risk of human pandemic influenza.” (pp. 6, 25) “…minimize the negative socio- economic impact…” (p. 25) “…minimize social disruption and economic burden.” (p. 26)	8 Some selected examples “…to minimize socio-economic and environmental impact.” (pp. 1, 43) “…minimize negative socioeconomic and environmental impact during pandemic” (p. 33) “…to minimize concern, social disruption, and stigmatization and correct misinformation.” (p. 61)	2 One selected example “To reduce the impact of the pandemic virus on morbidity and mortality and minimize social disruption minimize social disruption” (p. 16)	
9	Moral	1 “…bring in an element of moral hazard in compensation payments to organized poultry farms.” (p. 18)	-	-	-	-
10	Participation	11 Some selected examples “…with the participation of the Regional Directorates of Livestock Services and Regional Health Directorates and other governmental and non-governmental concerned organizations.” (pp. 4, 5)	1 “…with participation from relevant government, NGOs, private sectors…” (p. 25)	5 One selected example “…multi-sectoral approach with community participation and collaboration with International organizations.” (p. 1)	4 One selected example “Full mobilization of health services and strict enforcement of epidemic law during pandemic will only be successful on the basis of full participation of decentralized levels.” (pp. 21, 23)	
11	Protection	-	8 Some selected examples “…protection of healthcare workers and other vulnerable groups.” (p. 8) “…the protection and conservation of wildlife.” (p. 12)	6 One selected example “Strengthening safe clinical care with protection of health Personnel” (p. 45)	5 “This includes specific approaches…including protection of cullers and health care workers.” (p. 4)	
12	Reasonable/ Reasonableness	1 Reasonable care necessary (p. 172)	-	-	-	
13	Responsibility/Responsible/	38 Some selected examples “…ensuring responsible outbreak studying to avoid panic…” (p. 1) “…responsible media studying on avian influenza.” (p. 53)	2/27 One selected example “…pandemic preparedness is the responsibility of all…” (p. 6)	59 One selected example “The Forest Department (FD) is responsible for all activities concerning wildlife” (p. 16)	5 Some selected examples “Pandemic preparedness is the responsibility of all…” (p. 7) “…some agencies will bear the primary responsibility while the others will also be active…” (p. 15)	
14	Responsive/Responsiveness	-	1 “To establish and ensure an integrated communication strategy responsive to public concerns.” (p. 28)	-	1 “To establish and ensure an integrated communication strategy responsive to public concerns.” (p. 17)	
15	Right/Rights		1 “The constitution of the Peoples Republic of Bangladesh assures “health is the basic right of every citizen of the republic”.” (p. 26)	1 “The constitution of the People’s Republic of Bangladesh assures “health is the basic right of every citizen of the republic”.” (p. 37)	-	
16	Transparency/ Transparent	4 One selected example “…transparent and proactive public information strategy related to avian influenza and other epidemics.” (p. 53)	1 “Transparency is a key strategy to gain the public’s trust in the government and other stakeholders and is critical to disaster management.” (p. 8)	-	1 “Transparency is a key strategy to gain public trust in the government which is critical to disaster management.” (p. 4)	
17	Trust	5 Some selective examples “…communication failure by governmental officials could create panic among the public: undermine public trust/confidence…” (p. 16) “…maintain and restore trust.” (p. 49)	1 “Transparency is a key strategy to gain the public’s trust in the government and other stakeholders and is critical to disaster management.” (p. 8)	-	1 “Transparency is a key strategy to gain public trust in the government which is critical to disaster management.” (p. 4)	

## Appendix E

**Table A5:** Ethical terms in South Asian COVID-19 guidelines.

Sl. No.	Ethical Term/ Expression	Nepal (2021)	Bangladesh	Sri Lanka (2020)	India (2020)	Pakistan (2020)
1	Accountability	-	-	-	-	-
2	Collaboration/collaborative	6 One selected example “The plan envisioned collaborations between the public, non-governmental, and private sectors, as well as…” (p. 28)	-	2 An example “In collaboration with Ceylon College of Physicians”	-	3 One example “These decisions should therefore not be made alone but through a collaborative process which will help to share and lessen the burden.” (p. 6)
3	Communication	53 One selected example “The communication targets were set according to the socio-ecological model and epidemiology of the disease. It was also important for the messaging mechanisms to reach as wide an audience as possible” (p. 42)	1 “Communicate clearly: simple instructions; closed-loop communication (repeat instructions back); adequate volume without shouting” (p. 49)	5 Selected example “Availability of a dedicated smart phone and intercom facilities in cohort or triage ICU is important to improve communication and to prevent frequent staff movements.”	14 One example “Extensive inter-personal and community-based communication.” (p. 10)	3 “Compassionate, honest and direct communication with patients or surrogates is important beginning from the time of admission to the hospital.” (p. 6)
4	Equity	1 “The measures put in place as part of the COVID-19 response also took into consideration gender, equity, and human rights” (p. 44)	-	-		-
5	Ethic/s	5 One selected sample “Perceptions on Ethics of Public Health Interventions during the COVID-19 Outbreak” (p. 79)	-	-		29 One example “COVID-19 pandemic poses a catastrophic health emergency which necessitates prudent use of scarce resources while safeguarding ethical values and professional virtues that form the core of humanistic health care for patients.” (p. 4)
6	Fair/Fairness	-	-	-		-
7	Human Rights	1 “The measures put in place as part of the COVID-19 response also took into consideration gender, equity, and human rights” (p. 44)		-		-
8	Minimising harm/risk/negative impact/social disruption/economic impact	1 “Minimising the risk of transmission from the infected or suspected.” (p. 27)	-	11 Selected example “Areas separated from the rest of the ICU beds to minimize risk of in-hospital transmission” (p. 14)		1 “…is to provide an ethical framework for institutions to formulate Standard Operating Procedures for making healthcare decisions that will maximize benefits to the public while minimizing risks to healthcare providers” (p. 3)
9	Moral	2 “The Ministry of Health and Population (MoHP) provided NARTC with PPE sets, which included gowns, boots and masks, among others, for their frontline workers which was a huge boost to morale.” (p. 14)		-		1 “Allocation of limited resources can be morally distressing and emotionally draining for clinicians.” (p. 6)
10	Participation	1 “The new laboratory network was facilitated by the federal MoHP with the active participation and contribution of provincial and local governments and the private sector.” (p. 63)				-
11	Protection/Protect	20 One selected example “When the next pandemic strikes, the whole world needs to implement best practices to save lives and protect livelihoods.” (p. 89)	8 One example “Protecting healthcare providers is the first priority, as you are the primary line of defense for this patient, and upcoming patients.” (p. 48)	7 “Lung protective ventilator strategy remains the mainstay of delivering ventilator therapy.” (p. 18)	8 One example “Buffer stock of personal protective equipment maintained.” (p. 4)	5 One example “Dire circumstances of the pandemic necessitate shifting to a public health approach that requires distribution of scarce resources for the benefit and protection of the larger society often times at the expense of benefits to individual patients.” (p. 4)
12	Reasonable/ Reasonableness	-	4 One example “In medical wards not equipped with negative pressure rooms, like those which admit most COVID-19 patients because of reduced bed availability, it is reasonable to imagine a higher exhaled air dispersion and contamination.” (p. 29)	-		-
13	Representation/represent	17 One example “On January 26, 2020, Patan Hospital held a multispecialty meeting and formed a task force that consisted of representatives from various departments, each having specific roles and responsibilities.” (p. 12)		2 One example “Multi-disciplinary expert team composed of consultant microbiologist/Physician or any representative from the clinical team; e.g., Respiratory Physician, Intensivist, Virologist based on the availability (for overall technical guidance)” (p. 30)	1 “At the Central level, only Secretary (H) or representative nominated by her shall address the media” (p. 20)	-
14	Responsibility/Responsible	3 One example “The Nepal Health Research Council (NHRC) is the national autonomous apical body responsible for conducting and supporting health research with the highest level of ethical standards within the Republic of Nepal.” (p. 75)	1 “The referring team shall maintain primary responsibility for the patient with a multidisciplinary team approach to patient management.” (p. 56)	1 One example “The referring team shall maintain responsibility for the patient up to admission to ICU, and shall remain responsible for ongoing management if admission is refused or deferred.” (p. 13)	2 One example “Under which data managers (deployed from IDSP/NHM) responsible for collecting, collating and analysing data from field and health facilities.” (p. 21)	2 One example “It is the responsibility of institutions and government to ensure the availability of compassionate end of life care and appropriate personnel.” (p. 5)
15	Responsive/Responsiveness	-		-	-	-
16	Right/Rights	6 “The measures put in place as part of the COVID-19 response also took into consideration gender, equity, and human rights” (p. 44)		-	-	-
17	Transparency	2 One example “Consistency and transparency are vital in information sharing.” (p. 88)		-	-	1 “This may be through use of institutional notice boards or websites to ensure public awareness and transparency” (p. 4)
18	Trust	7 One example “To engage the public and increase compliance, respected personalities should be brought in to build community trust.” (p. 23)		-	-	2 One example “Trust is the core of ethical physician–patient relationships.” (p. 6)
